# Reduced brain activation during imitation and observation of others in children with pervasive developmental disorder: a pilot study

**DOI:** 10.1186/1744-9081-9-21

**Published:** 2013-05-29

**Authors:** Aiko Kajiume, Shiori Aoyama-Setoyama, Yuri Saito-Hori, Nobutsune Ishikawa, Masao Kobayashi

**Affiliations:** 1 Department of Pediatrics, Hiroshima University Graduate School of Biomedical & Health Sciences, 1-2-3 Kasumi, Minami-ku, Hiroshima, 734-8551, Japan; 2 Department of Child Studies, Sendai Seiyo Gakuin collage, 3-5-75 Itsutsubashi, Wakabayashi-ku, Sendai, 984-0022, Japan

**Keywords:** Pervasive developmental disorder, Imitation, Mirror neuron systems, Near-infrared spectroscopy

## Abstract

**Background:**

Children with pervasive developmental disorder (PDD) are thought to have poor imitation abilities. Recently, this characteristic has been suggested to reflect impairments in mirror neuron systems (MNS). We used near-infrared spectroscopy (NIRS) to examine the brain activity of children with PDD during tasks involving imitation and observations of others.

**Findings:**

The subjects were 6 male children with PDD (8–14 years old) and 6 age- and gender-matched normal subjects (9–13 years old). A video in which a woman was opening and closing a bottle cap was used as a stimulus. Hemoglobin concentration changes around the posterior part of the inferior frontal gyrus and the adjacent ventral premotor cortex were measured with a 24-channel NIRS machine during action observation and action imitation tasks. Regional oxygenated hemoglobin concentration changes were significantly smaller in the PDD group than in the control group. Moreover, these differences were clearer in the action observation task than in the action imitation task.

**Conclusions:**

Dysfunction in the MNS in children with PDD was suggested by the reduced activation in key MNS regions during tasks involving observations and imitations of others. These preliminary results suggest that further studies are needed to verify MNS dysfunction in children with PDD.

## Background

Recent functional brain imaging studies of patients with pervasive developmental disorder (PDD) have indicated that abnormalities in recognition cause various PDD symptoms [1]. In addition, “the broken mirror theory of autism” [2,3] has been proposed after the discovery of mirror neuron systems (MNS). Mirror neurons, which were discovered in monkey F5 and PF areas, are activated when a monkey performs an action and observes it [4,5]. These areas are assumed to correspond to the human inferior frontal gyrus (IFG) and inferior parietal lobule [6], respectively, and functional magnetic resonance imaging (fMRI) and electroencephalography (EEG) studies have suggested that these areas contain human mirror neurons [7,8]. Several studies have used various brain imaging techniques to test this hypothesis e.g., [9,10]. However, their results have been controversial. Therefore, further evidence is needed.

Among the many brain imaging techniques, we adopted near-infrared spectroscopy (NIRS) for the following reasons. First, compared to other brain imaging techniques, subjects can move somewhat, and their brain activation is measured in a more natural state. This is important when studying children with PDD because they are hyperkinetic and easily anxious in response to background noise or obstructive spaces.

Therefore, we conducted a NIRS study to explore MNS dysfunction in children with PDD during action-observation and action-imitation tasks. We enrolled only boys because patients with PDD are predominantly male and data collected from girls might affect the results. We predicted that children with PDD would exhibit smaller oxygenated hemoglobin concentration ([oxy-Hb]) changes compared to normal subjects.

## Methods

### Subjects

The subjects were 6 right-handed children with PDD (boys; 8–14 years old; mean, 10.7 years; SD, 2.9 years) and 6 age- and gender-matched normal control subjects (boys; 9–13 years old; mean, 10.9 years; SD, 1.6 years) (Table 1). All children with PDD were outpatients of the Department of Pediatrics, Hiroshima University Hospital, and their conditions were diagnosed according to the criteria of the Diagnostic and Statistical Manual of Mental Disorders, fourth edition. None of the subjects used psychotropic drugs. Normal subjects were recruited through the homepage of the Department of Pediatrics, Hiroshima University. The “social skill test for students [11],” a rating scale often used to assess the social skills of students, was administered to the subjects’ parents. This test assesses 4 areas: collective behavior, self-control skills, peer relationships, and communication skills. Ten points in each subscore are equivalent to the age average. The intelligence quotients of the children with PDD were determined with the Wechsler Intelligence Scale for Children, third edition [12]. This study was approved by the Ethical Committee of Clinical Study, Hiroshima University Hospital, and written informed consents were obtained from all subjects’ parents before the subjects participated in the study.

**Table 1:** Clinical and demographic details of the subjects

	**Age**	**Diagnosis**	**IQ**	**Social skill test**			
				**Collective behavior**	**Self control skills**	**Peer Relationships**	**Communication skills**
PDD							
PDD1	8.1	PDDNOS	88	6	7	10	7
PDD2	9.6	Asperger	118	4	5	5	5
PDD3	8.8	PDDNOS	115	6	9	8	10
PDD4	11.2	Asperger	110	4	7	3	8
PDD5	15.3	PDDNOS	85	2	1	7	3
PDD6	10.5	Asperger	118	5	6	5	8
Mean	10.7		105.8	4.5^**^	5.8^*^	6.3^**^	6.8^*^
S.D.	2.9		14.9	1.5	2.7	2.5	2.5
Control subjects (n = 6)							
Mean	10.9			10.8	10.2	12.3	11.2
S.D.	1.6			2.3	1.0	1.5	2.9

*IQ* intelligence quotients, *PDDNOS* Pervasive developmental disorder not otherwise specified.

^*^p < 0.05; ^**^p < 0.01.

### Activation tasks

**Figure 1:**
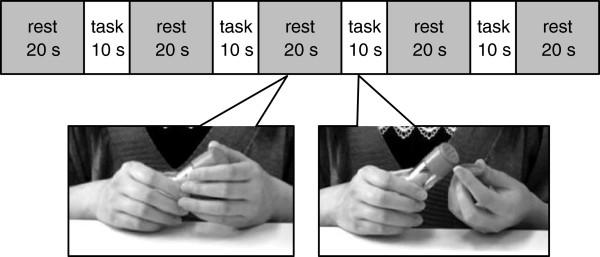
Experimental protocol of the action observation and action imitation tasks.

We made a video in which a woman opens and closes a bottle with a one-touch cap. In the video, she holds the bottle during rest periods and opens and closes the bottle during activation periods. The activation periods were 10 s long, and they were followed by a 20-s rest (block design); this was repeated 4 times. Trials began and ended with a 20-s rest period (Figure 1).

The subjects sat on a chair in front of a 19-inch monitor. In the action-observation task, they only watched the video. In the action-imitation task, the subjects watched the video and imitated her movement. During the rest period, the subjects sat still.

### NIRS measurement

**Figure 2:**
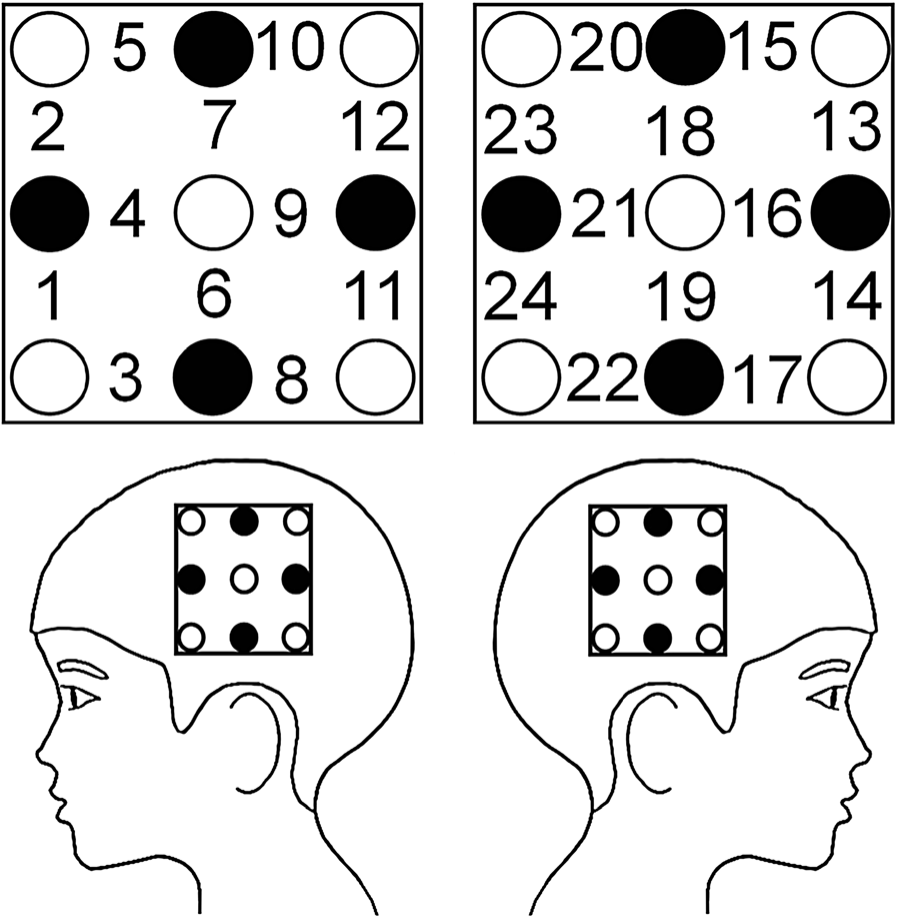
Probe positions and channel numbers. emitters, open circles; detectors, filled circles.

[Oxy-Hb] and deoxygeneted-Hb ([deoxy-Hb]) concentration were measured with a 24-channel NIRS machine (ETG-100; Hitachi Medical Corporation, Tokyo, Japan) at 2 wavelengths of near-infrared light (760 and 840 nm). The distance between the pairs of emission and detection probes was 3.0 cm. The NIRS machine can measure [Hb] changes in a depth of approximately 2–3 cm (i.e., only the cortical surface area). The changes in [oxy-Hb] and [deoxy-Hb] were calculated from the difference in the light absorption characteristics of these chromophores according to the Beer–Lambert law. The absorption of near-infrared light was measured with a time resolution of 0.1 s. Eighteen probes (10 emitters and 8 detectors) were set in 2 probe holders. The holders were placed on the head with the low central probe being positioned at the T3 or T4 electrode position of the International 10/20 system used in EEG [13] (Figure 2). The correspondence between the NIRS channels and locations in the cerebral cortex has been previously confirmed by a multi-subject study of anatomical craniocerebral correlation in adults [14], and the T3 and T4 placements corresponded to the bilateral middle temporal gyri with 94–96% probability.

### Statistical analysis

The [Oxy-Hb] data were analyzed because they are considered the most sensitive [15,16]. Channels 1, 2, 11–14, 23, and 24 (i.e., the most anterior and posterior channels of each probe set), in which low signal-to-noise ratios were observed with the exceptional channel function in the NIRS system, were excluded from further analysis.

The data were analyzed with the “integral mode” in the NIRS system to adjust the [oxy-Hb] data for long-term oscillations that were not due to activation tasks. The pre-task baseline was determined as the mean value during the 5-s period just before the activation period, and the post-task baseline was determined as the mean value during the last 5 s of the post-task period. Moving average methods were used to exclude short-term motion artifacts in the analyzed data (moving average window, 5 s). The grand average waveforms were calculated with “multiple data analysis” in the NIRS system.

Next, we determined the [oxy-Hb] changes in each channel by calculating the differences in the means from 2.5 to 7.5 s of the activation period and just before 5 s of the activation period in order to quantify the neural-induced concentration changes (hemodynamic response). The [oxy-Hb] changes in each channel were analyzed with a two-way repeated-measures analysis of variance (ANOVA) with “task” (i.e., the action observation and action imitation tasks) as the intra-individual independent variable and “diagnosis” (i.e., PDD and normal control) as the inter-individual independent variable. This ANOVA was exploratory because the subject number was too small to completely justify parametric analysis methods. The level of significance was set at *P* < 0.05. For factors that exhibited significance, a post hoc *t*-test was performed; significance levels were corrected by the false-discovery rate method [17].

## Results

All social skill scores were significantly lower in the PDD group compared to the control group (Table 1). Although the PDD group included patients with PDD not otherwise specified and Asperger disorders, these subgroups did not significantly differ for sex, age, social skill scores, and [oxy-Hb] changes during the action observation and action imitation tasks.

**Figure 3:**
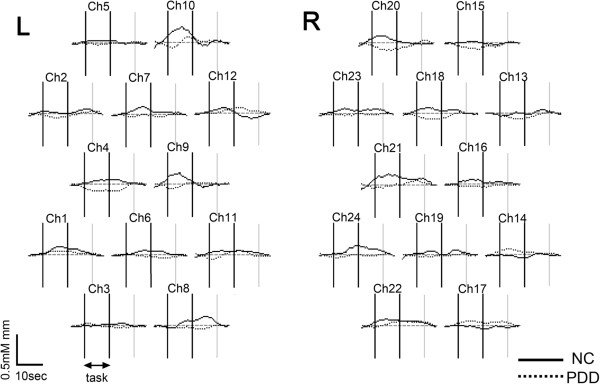
**Grand average waveforms of oxygenated hemoglobin concentration changes during the action observation task for controls (*N*** **= 6) and PDDs (*N = 6).*** Controls, solid line; PDD, broken line.

**Figure 4:**
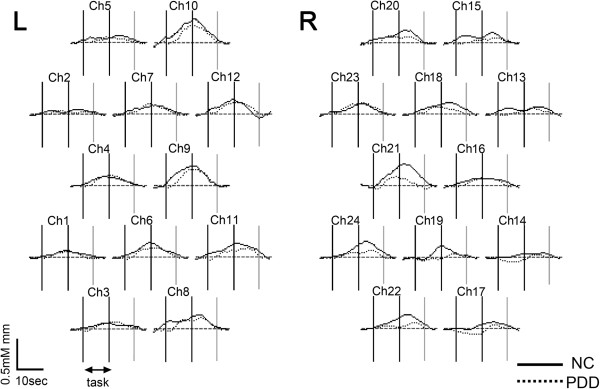
**Grand average waveforms of oxygenated hemoglobin concentration changes during the action imitation task for controls (*N*** **= 6) and PDDs (*N = 6).*** Controls, solid line; PDD, broken line.

For the [oxy-Hb] changes, the two-way repeated-measures ANOVA revealed a significant main effect of “diagnosis” in 5 channels (channels 7, 15, 18, 21, and 22; *F*, 7.3–14.2, *P* < 0.014) with the significance levels corrected by the false-discovery rate method. The [oxy-Hb] changes were significantly larger in the control group than in the PDD group, that is, more increasing or less decreasing. The two-way interaction of task by diagnosis did not exhibit statistical significance after correction by the false-discovery rate method, but we found clearer differences between the groups in the action-observation task than in the action-imitation task (Figures 3 and 4). The post-hoc *t*-test demonstrated significantly smaller [oxy-Hb] increases or larger decreases in the PDD group than in the control group in 5 channels (channels 7, 15, 18, 21, and 22) during the action observation task, and in 1 channel (channel 15) during the action imitation task in the PDD group than in the control group. Hence, the differences were largely lateralized to the right.

## Discussion

In this preliminary study, we used NIRS to examine the brain function of children with PDD during tasks involving the observation and imitation of others. The results showed that cortical activation during these tasks was significantly smaller in the PDD group than in the control group, and the main differences were on the right hemisphere. We placed the 18 probes so as to cover the posterior part of the IFG and the adjacent ventral premotor cortex (PMC) regions, where human MNS are located [18]. Thus, the channels with significant differences were thought to be relevant to the parts of the MNS. Some studies have shown that mirror neurons function on both sides. Therefore, we placed the probes on both sides of the head. Although the main differences were on the right hemisphere, the channels on the left also showed differences between the groups (e.g., channels 8 and 10). Because this study is preliminary, we need more subjects to study laterality.

Next, the significant differences between the two groups were clearer in the action-observation task compared to the action-imitation task. This difference may have been influenced by goal-oriented imitation [19], and not automatic imitation, which is impaired in PDD patients. In addition, the consciousness of others, which is weak in PDD children, plays an important role in imitation [20]. With more subjects, we need to explore the differential brain activation between action-observation and imitation tasks.

Our study had several limitations. First, the sample size was small. We should increase the subjects to clarify the validity of this result in the future. Second, the PDD group included subjects with heterogeneous diagnoses. We need to assess differences with respect to diagnoses with more subjects. Third, subject head sizes were smaller than those in past studies of the correspondence between NIRS channels and locations. Other modalities, such as 3-dimensional MRI, could be used in combination with NIRS to precisely analyze the anatomical locations that are activated.

## Conclusion

In this pilot study, brain activation during action-observation and action-imitation tasks was clearer in the control group than in the PDD group, particularly on the right hemisphere. Further studies are needed to verify the results of this preliminary study and to investigate the mechanisms that underlie the poor imitation abilities of children with PDD.

## Ethical approval

This study was approved by the Ethical Committee of Clinical Study, Hiroshima University Hospital, and written informed consent was obtained from all subjects’ parents before their participation in the study.

## Competing interests

All the authors declare that they have no competing interests with respect to this study or its publication.

## Author’ contributions

AK designed the study, wrote the protocol, collected the data, statistically analyzed the data, and wrote the first draft of the manuscript. YSH and SAS were involved in working out the study design and data collection, and contributed to writing the final version of the manuscript. NI was involved in analyzing the data and writing the manuscript. MK is the head of our laboratory and contributed to writing the final version of the manuscript. All authors contributed to and have approved the final manuscript.
